# Differences in body composition and physical fitness parameters among prepubertal and pubertal children engaged in extracurricular sports: the active health study

**DOI:** 10.1093/eurpub/ckac075

**Published:** 2022-08-26

**Authors:** Samuel Manzano-Carrasco, Jorge Garcia-Unanue, Jorge Lopez-Fernandez, Antonio Hernandez-Martin, Javier Sanchez-Sanchez, Leonor Gallardo, Jose Luis Felipe

**Affiliations:** IGOID Research Group, Physical Activity and Sport Sciences Department, Faculty of Sport Sciences, University of Castilla-La Mancha, Toledo, Spain; IGOID Research Group, Physical Activity and Sport Sciences Department, Faculty of Sport Sciences, University of Castilla-La Mancha, Toledo, Spain; IGOID Research Group, Physical Activity and Sport Sciences Department, Faculty of Sport Sciences, University of Castilla-La Mancha, Toledo, Spain; School of Sport Sciences, Universidad Europea de Madrid, Madrid, Spain; IGOID Research Group, Physical Activity and Sport Sciences Department, Faculty of Sport Sciences, University of Castilla-La Mancha, Toledo, Spain; School of Sport Sciences, Universidad Europea de Madrid, Madrid, Spain; IGOID Research Group, Physical Activity and Sport Sciences Department, Faculty of Sport Sciences, University of Castilla-La Mancha, Toledo, Spain; School of Sport Sciences, Universidad Europea de Madrid, Madrid, Spain

## Abstract

**Background:**

This study aimed to analyze the associations of maturity status, chronological age and sex with physical fitness and body composition among active children.

**Methods:**

A total of 1682 children (72% boys; age = 11.22 ± 2.64 years; height = 147.57 ± 15.87 cm; weight = 44.55 ± 15.29 kg) from rural areas participating in extracurricular sports were divided into four groups according to their sex and maturity status (prepubertal and pubertal according to stages described by Tanner). Body composition (body mass index, muscle mass and fat mass) and physical fitness (20-m shuttle-run test, handgrip strength and vertical jump) were assessed using standardized procedures. A two-way ANOVA and product–moment correlations were performed.

**Results:**

Prepubertal boys had more fat mass (%) than pubertal boys [*P*<0.001; effect size (ES): 0.45], while prepubertal girls had more muscle mass (%) than pubertal girls (*P*<0.001; ES: 0.47). The pubertal group displayed higher fitness outcomes (absolute values) regardless of sex (*P*<0.05). However, the prepubertal group had higher percentile values in the 20-m shuttle-run test and vertical jumps than the pubertal group regardless of sex (*P *<* *0.001; ES: 0.29–0.48). All the measures of physical fitness were positively associated with chronological age and muscle mass (%).

**Conclusions:**

Although absolute values of body composition and physical fitness appear to increase among pubertal children participating in extracurricular sports, the percentiles indicate that puberty is accompanied with a loss of a physical fitness levels. Thus, extracurricular sports might not be enough to enhance fitness among adolescents.

## Introduction

Biological maturation is the process of progressing towards a mature state, and can be defined in terms of status (specific stage or extent of change), tempo (rate of change) and maturity timing (onset of change).^1^ Depending on the person, the physical and physiological changes that occur within a stage evolve at a different rate.^1^ Many physical and physiological changes occur during childhood and adolescence; these are crucial periods of life with respect to the establishment of lifestyle habits.^2^ Childhood and adolescence are also characterized by sexually dimorphic changes in body size and composition, as well as morphological and functional changes in cardiorespiratory, metabolic and neuromuscular systems.^3^^,^^4^ Consequently, during these stages modifiable parameters, such as body composition, physical activity (PA) or physical fitness (PF), are important to track, as they will have a significant impact on the quality of life later in adulthood.^5^

Children of the same chronological age can differ significantly in maturity as some individuals mature earlier or later than their peers.^6^ The main difference occurs between boys and girls; skeletal maturity (i.e. higher biological age) occurs earlier in girls than boys,^7^ and leads to different adaptations. For instance, boys show a greater tendency to gain lean mass while girls accumulate more body fat.^8^ There are also differences in body fat distribution; boys tend to accumulate fat around the waist, and girls tend to accumulate fat peripherally and on the hips.^9^ For this reason, anthropometric and body composition differences amongst youths in this period can be controlled and explained through the assessment of maturity status.^10^

Biological maturation and body development are two factors that, together with PA levels, influence PF. A combination of different physiological and psychological parameters necessary for daily activities is considered as constituting PF.^11^ The main factors of health-related PF include cardiorespiratory, musculoskeletal and motor fitness.^12^ PF is generally recognized as a key determinant of current and future health status in both childhood and adulthood.^12^^,^^13^ Evidence has shown that changes in PF and body composition occur as a result of the maturation process.^14^ However, to achieve higher PF and healthy body composition, adolescents have to participate in regular PA.^15^ Accordingly, an active lifestyle improves children and adolescents’ metabolic, musculoskeletal, psychosocial, cardiac and cognitive health.^16^^,^^17^ Despite the health-related benefit of PA and its relationship with PF, it is estimated that 81% of young Europeans do not engage in sufficient daily PA.^18^ Participation in extracurricular structured sports after school is a common formula used by rural public organizations for improving the PA levels of children and adolescents.^15^ Newer evidence suggests that adolescents enrolled in extracurricular sports activities show low levels of PF or poor body composition values,^19^ probably due to a combination of unhealthy behaviours, such as lack of engaging in 60 min of moderate to vigorous PA every day, excessive screen time, or sitting time and higher energy intake.^20^ Nonetheless, further research is needed to confirm this evidence and to understand the current habits of the young rural population participating in extracurricular sports, and whether these changes according to age and sex. Therefore, using a cross-sectional study design, this study aimed to investigate the influence of maturity status, chronological age and sex on PF and body composition in a young population participating in extracurricular sports.

## Methods

### Study design and sample

A cross-sectional study was performed with rural participants aged 6–17 years (age = 11.22 ± 2.64 years; height = 147.57 ± 15.87 cm; weight =44.55 ± 15.29 kg). The study was conducted with a convenience sample enrolled in different municipal sports schools during the 2018–20 school year in Castilla-La Mancha (a region located in the centre of Spain). All participants practised an extracurricular sports activity (e.g. football, basketball, tennis, volleyball, zumba, etc.) at least 2 days a week for 1 h each day. Participants and their parents or legal tutors were informed of the research objectives and test characteristics before the study began. They were required to give their informed consent prior to their son and/or daughters’ participation in the testing.

The final study sample was comprised of 1682 children and adolescents (72% boys and 28% girls) and was divided into four subsamples depending on sex (boys and girls) and maturity status (prepubertal and pubertal). Given the significance of maturity in this population, a Marshall and Tanner test^21^ was performed using a self-assessment questionnaire with illustrations (with Stage I categorized as prepubertal, and Stages II and III as pubertal). The final four subsamples comprised 792 prepubertal boys, 419 pubertal boys, 348 prepubertal girls and 123 pubertal girls (table 1). All participants were individually assessed prior to a training session of the sport in which they were enrolled. The test lasted between 60 and 90 min, and was conducted in groups of 12–14 participants. PF and body composition tests were administered by qualified personnel and according to the protocol established in the ‘Active Health’ project. In addition, the PF were administered in such a way that fatigue did not influence the performance of the subsequent assessments.

**Table 1:** Descriptive characteristics of the participants and body composition and PF variables

	Boys (*n *=* *1211)		Girls (*n *=* *471)	
Variables	Prepubertal	Pubertal	Prepubertal	Pubertal
	(*n *=* *792)	(*n *=* *419)	(*n *=* *348)	(*n *=* *123)
Age (years)	9.84 ± 1.75	14.35 ± 1.11	9.56 ± 1.79	14.08 ± 1.01
Weekly sports practice (day/week)	3.20 ± 1.53	4.16 ± 2.38	2.74 ± 1.73	3.76 ± 2.53
Sports practice (years)	3.92 ± 2.10	6.13 ± 3.20	3.00 ± 1.76	4.46 ± 2.99
Weight (kg)	38.73 ± 11.56	59.00 ± 13.65	36.58 ± 11.40	55.41 ± 10.48
Height (cm)	140.64 ± 11.75	165.18 ± 9.62	138.13 ± 12.58	159.02 ± 6.68
BMI (kg/m^2^)	19.22 ± 3.55^a^^,^^b^	21.45 ± 3.74	18.75 ± 3.40^b^	21.87 ± 3.76
Total fat mass (kg)	9.64 ± 5.51^b^	12.69 ± 6.39^a^	9.95 ± 4.99^b^	16.51 ± 6.38
Fat mass (%)	23.60 ± 6.62^a^^,b^	20.65 ± 6.31^a^	26.13 ± 5.50^b^	28.94 ± 5.96
Total muscle mass (kg)	27.52 ± 6.78^a^^,b^	43.91 ± 8.49^a^	25.24 ± 6.71^b^	36.92 ± 4.98
Muscle mass (%)	72.23 ± 6.20^a^^,b^	75.26 ± 5.96^a^	69.99 ± 5.18^b^	67.42 ± 5.65
20 mSRT (stages)	4.97 ± 2.02^a^^,^^b^	7.64 ± 2.20^a^	3.84 ± 1.60^b^	4.94 ± 1.64
20 mSRT (pc)	66.26 ± 24.04^a^^,^^b^	59.20 ± 24.08	73.61 ± 22.56^b^	61.60 ± 26.71
Handgrip (kg)	18.08 ± 6.18^a^^,^^b^	32.68 ± 9.27^a^	16.12 ± 5.50^b^	24.67 ± 4.89
Handgrip (pc)	53.82 ± 28.90^b^	49.55 ± 29.39	53.52 ± 30.93	47.75 ± 27.57
CMJ (cm)	20.79 ± 4.93^a^^,^^b^	31.19 ± 6.40^a^	19.71 ± 4.61^b^	25.25 ± 5.25
CMJ (pc)	48.76 ± 26.49^b^	32.86 ± 19.93	50.79 ± 26.07^b^	39.22 ± 23.93

Data are presented as mean ± standard deviation in continuous variables. BMI, body mass index; 20 mSRT, 20-m shuttle-run test; Handgrip, handgrip strength; CMJ, countermovement jump; pc, percentile; kg, kilograms; cm, centimetres.

a The difference in means between each sex group in each maturity stage is significant at the 0.05 level.

b The difference in means between maturity stage in each sex group is significant at the 0.05 level.

The study was conducted according to the standards of the Declaration of Helsinki (2013 revision, Brazil) and the European Community Guidelines for Good Clinical Practice (111/3976/88 July 1990). The instructions for clinical research in humans governed by the Spanish legal framework were followed (Royal Decree 561/1993). The Bioethics Committee for Clinical Research of the Virgen de la Salud Hospital in Toledo (Spain) approved the ‘Active Health’ project (Ref.: 508/17042020).

### Procedures and measurements

#### Anthropometric measurements

A portable segmental analyser of multifrequency body composition (Tanita MC-780, Tanita Corp., Tokyo, Japan) was used to measure weight (kilograms), fat mass (kilograms and %), and muscle mass (kilograms and %). Height (centimetres) was assessed with a height rod (Seca 214, Hamburg, Germany). Body mass index (BMI) was calculated by dividing weight (kilograms) by squared height (metres). The evaluations were carried out barefoot and with clothes.

#### Physical fitness

The different parameters of PF were measured using an adapted version of the extended Assessing Levels of Physical Activity health-related fitness battery, which is valid, feasible, reliable and safe for the assessment in children and adolescents.^22^ The components are explained below:

Cardiorespiratory fitness was assessed by performing a maximum incremental field test [20-m shuttle-run test (20 mSRT)]. This valid and reliable field test is commonly used in studies involving children and adolescents.^11^ Participants had to run between two lines 20 m apart while keeping a pace emitted by acoustic signals in a portable speaker. The initial speed was 8.5 kmh^−^^1^, and this was increased by 0.5 kmh^−^^1^ each minute.^23^ The test ended when the participant failed to reach the lines concurrent with the audio signals on two consecutive occasions or when participants stopped because of fatigue. The results were transformed into stages of 1-min duration. The test was performed only once at the very end of the data collection fitness measures, so that performance and fatigue did not interfere with the results of the other tests. Percentile values based on age and sex were used to standardize the test results.^24^^,^^25^

An electronic hand dynamometer with adjustable grip was used to evaluate upper-body muscular strength (Constant R Model: 14192-709E). Participants in a full extension elbow position had to close their hand with continuous maximum force for 2 s. The test was alternately performed with the dominant hand and the non-dominant hand. Two attempts were allowed with 30 s of recovery in between. The best score from the dominant hand of each participant was taken into account for an analysis to the nearest 1 g, and was recorded in kilograms.^22^

A countermovement jump test (CMJ) with a free swing of the arms known as an abalakov jump was completed to assess lower-body muscular strength. This test is reliable and valid to assess lower-body muscular power in the young population.^26^ Participants were instructed to jump as high as possible and were allowed three attempts with 1 min of recovery between each attempt. A pair of parallel bars with photoelectric cells was used (Optojump, Microgate, Bolzano, Italy) to measure flight time, i.e. the duration between take-off and landing. Height of jump was recorded in centimetres and calculated to the nearest 0.1 cm. Percentile values based on age and sex were also used to standardize the test results.^24^^,^^25^

#### Statistical analysis

Means and standard deviations were presented for all quantitative variables. Firstly, the Kolmogorov–Smirnov test was used to confirm the normal distribution of the data. Differences between groups were evaluated through two-way ANOVA (boys vs. girls; prepubertal vs. pubertal). All anthropometric and PF variables were used as dependent variables. Maturity status and sex were used as fixed factors. The Bonferroni *post hoc* test was carried out to determine the differences amongst groups. Product–moment correlations were performed to evaluate the relationship of chronological age in months and PF variables and the relationship between normalized anthropometric and PF variables. *Z* scores were used to normalize the main anthropometric and PF variables independently for each age and sex group. All data were statistically analyzed using SPSS Version 24.0 for Windows (IBM Corp, Chicago, IL). The level of significance was set at *P* < 0.05.

## Results

The differences in body composition variables, separated by sex (boys and girls) and maturity stage (prepubertal and pubertal) are shown in table 1. Significant differences in all body composition variables were found among the four subgroups. Pubertal group showed higher BMI, fat mass (kilograms) and muscle mass (kilograms) than their prepubertal peers in both sexes [*P* < 0.05; effect size (ES): 0.51–2.14]. Prepubertal boys showed significantly higher BMI and muscle mass (kilograms) values compared with prepubertal girls (*P* < 0.001; ES: 0.13–1.03). By contrast, no significant differences were found in the fat mass (kilograms) of prepubertal boys compared with prepubertal girls (*P* = 0.40; ES: 0.05). Pubertal girls showed higher fat mass values (kilograms) compared with pubertal boys (*P* < 0.001; ES: 0.59). Conversely, pubertal boys had more muscle mass (kilograms) compared with pubertal girls (*P* < 0.001; ES: 1.03). No significant differences were found in the BMI of boys and girls in the pubertal stage (*P* = 0.25; ES: 0.11).

On the other hand, prepubertal boys had higher values than pubertal boys in percentage of fat mass (*P* < 0.001; ES: 0.45). However, pubertal boys had higher muscle mass (%) than prepubertal boys (*P* < 0.001; ES: 0.49). In girls, the prepubertal group had a lower percentage of fat mass than pubertal girls (*P* < 0.001; ES: 0.49) but higher muscle mass (%) values than pubertal girls (*P* < 0.001; ES: 0.47). Finally, boys had significantly higher muscle mass (%), but lower fat mass than girls in both maturity groups (*P* < 0.001; ES: 0.39–1.35).

Table 1 also shows the differences in PF parameters separated by sex and stage of maturity. The pubertal group performed significantly better in the 20 mSRT (stages), handgrip strength (kilograms) and CMJ (centimetres) than the prepubertal group in both sex groups (*P* < 0.05; ES: 0.67–1.88). In addition, the prepubertal group revealed higher values in the 20 mSRT (percentiles) and CMJ (percentiles) than the pubertal group in each sex group (*P *<* *0.001; ES: 0.29–0.48). Also, the prepubertal boys’ group showed higher values in the handgrip test (percentile) than the pubertal group (*P* < 0.05). Nonetheless, no significant differences were found in the prepubertal girls’ group in the handgrip test percentiles (*P* = 0.875; ES: 0.19). Boys reported significantly higher values in 20 mSRT (stages), handgrip (kilograms) and CMJ (centimetres) than girls in both maturity groups (*P* < 0.05; ES: 0.22–1.40). By contrast, prepubertal girls showed higher values than prepubertal boys (*P* < 0.001; ES: 0.31) only in the 20 mSRT (percentiles). No significant differences were observed in the percentile of handgrip and CMJ tests between any group (*P* > 0.05; ES: 0.01–0.29).

**Figure 1:**
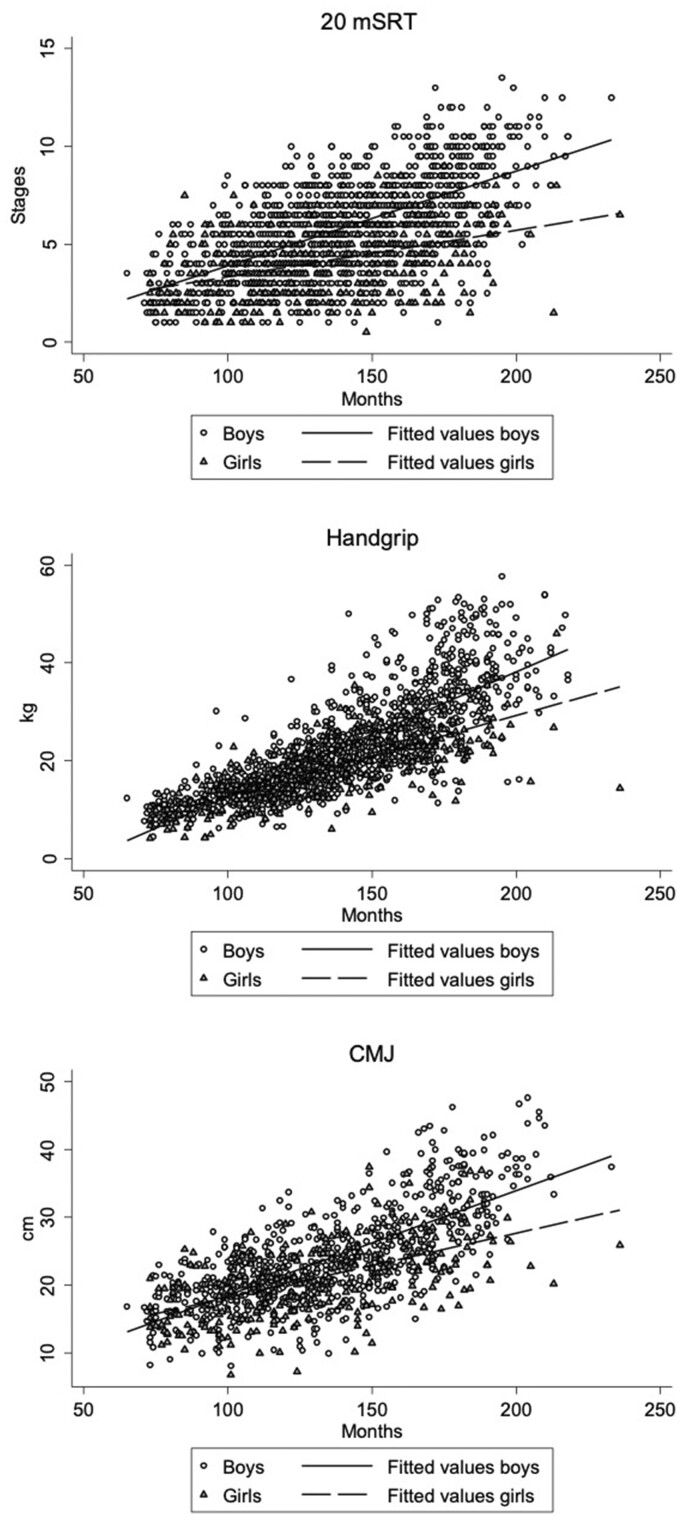
Relationship between age and average performance in PF tests (20 mSRT, handgrip strength and CMJ) for boys and girls aged 6–17*.* Note: observed values are plotted using triangles and circles for boys and girls, respectively. Solid and dashed lines display the predicted values of the linear regression models in the boys’ and girls’ groups, respectively

The correlations between chronological age in months and performance in PF variables (figure 1) showed a positive relationship in boys (*r *=* *0.61 for 20 mSRT in stages, *r *=* *0.79 for handgrip in kilograms and *r *=* *0.73 for CMJ in centimetres) and girls, although the correlation was less robust for girls (*r *=* *0.24 for 20 mSRT in stages, *r *=* *0.45 for handgrip in kilograms and *r *=* *0.56 for CMJ in centimetres).

**Figure 2:**
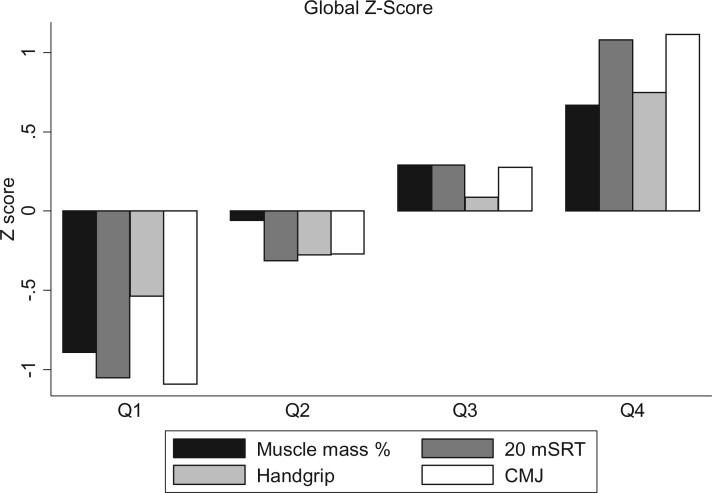
Global *Z* score. Note: the quartiles are calculated by the summing of the four *Z* scores for each variable. The bars show the mean value of each *Z* score in this quartile

There was a positive and significant relationship between all the variables of PF and muscle mass (%), normalized in *Z* scores (table 2). The only negative correlation, albeit with a very low coefficient, was between the percentage of muscle mass and handgrip strength. As figure 2 shows, the different parameters that were added to form the global *Z* scores revealed a similar pattern, with negative values in Quartiles 1 and 2, and positive values in Quartiles 3 and 4.

**Table 2:** Correlation between the normalized variables of body composition and PF

	*Z* score	*Z* score	*Z* score (handgrip)	*Z* score
	(muscle mass %)	(20 mSRT)		(CMJ)
*Z* score (muscle mass %)	1	0.489^a^	−0.144^a^	0.454^a^
*Z* score (20 mSRT)		1	0.235^a^	0.613^a^
*Z* score (handgrip)			1	0.386^a^
*Z* score (CMJ)				1

20 mSRT, 20 m shuttle-run test; Handgrip, handgrip strength; CMJ, countermovement jump.

a *P* < 0.01.

## Discussion

This study investigated the influence of maturity status, chronological age and sex on PF and body composition in 1682 children and adolescents from rural areas participating in extracurricular municipal sports. The main findings were that the absolute values of both body composition and PF parameters improved with advanced pubertal stage, regardless of sex. However, with regard to percentiles, PF decreased from prepuberty to puberty. Finally, PF variables were positively correlated with chronological age and muscle mass (%) regardless of sex. For this reason, it may be possible to standardize PF variables, and integrate them into a general or global health indicator.

Body composition and health in adulthood can be preceded by anthropometric changes and PA levels occurring during childhood and adolescence.^27^^,^^28^ For instance, aligned with previous studies, a significant increase in body height and mass occurs with increasing maturity and chronological age.^29^ However, variables, such as PA behaviour might influence changes in adolescents’ body composition during puberty.^30^ Accordingly, extracurricular sports might positively influence body composition variables in the step from prepuberty to puberty. This is evidenced in this study as the differences found in BMI, fat mass (kilograms and %) and muscle mass (kilograms and %) either in boys or girls engaged in extracurricular sports. However, the methodology of this study does not permit conclusions with regard to cause–effect. The sex comparison revealed that girls engaged in extracurricular sports display higher fat mass and lower muscle mass than boys in both maturity stages. This may be because the girls are often less active than the boys throughout the day.^31^ Further research is needed to control and monitor participants’ PA levels, as during puberty girls accumulate a higher accumulation of body mass during puberty^32^ while boys tend to improve muscle mass due to an increase in testosterone.^33^ Nonetheless, despite these changes in body composition during puberty, current results revealed no significant differences in BMI between pubertal boys and girls, suggesting that this indicator might not be accurate enough to determine body composition. In fact, a recent study suggests the maturation process is statistically related to BMI in boys but not in girls.^32^

Scientific evidence has shown that every element of PF has a positive effect on the health status of young people.^34^^,^^35^ Moreover, high levels of cardiorespiratory fitness, along with muscular strength and good healthy composition during childhood and adolescence, are related to better cardiovascular parameters and a lower risk of death in adulthood.^12^^,^^36^ In this study, the pubertal group showed higher performance in all tests compared to both prepubertal groups. These findings align with a previous study in which athletes with higher maturity offset performed significantly better in various PF tests compared with those athletes with lower values.^29^ Accordingly, some of the consequences of maturation (e.g. increase in body size, hormones, etc.) are related to the improvements in various PF components and muscle strength.^37^ Understanding how PA habits influence changes in PF in the transition from prepuberty to puberty is paramount to improving the health of adolescents and adults. When analyzed by percentile of the 20 mSRT test, prepubertal boys and girls were more fit than pubertal boys and girls, despite pubertal boys displaying higher outcomes. This fact might be due to PA levels seeming to decrease with age, and adolescents engaging in lower PA than children. Furthermore, pubertal adolescents also seem to accumulate more daily sitting time than prepubertal children.^38^ Therefore, although further research is needed, this study provides evidence that extracurricular sports activities and physical education classes might not be enough to maintain PF levels of children during the adolescence. Additionally, the participants in this study displayed lower performance in the PF test than the reference sample from the study of Gulías-González et al.^25^ This means that despite participating in extracurricular sports, today’s young population are less physically fit than those studied in 2014. Thus, despite the limitations of this study, our findings support the inclusion of PF components in national monitoring surveillance to better understand the effectiveness of PA policies.

Finally, this study evidence indicates that all measures of PF had a positive relationship with chronological age and muscle mass (%), regardless of sex. Accordingly, although boys and girls show similar performance in the prepubertal stage, boys increase their performance in PF tests compared to the girls as the stage of maturation increases. In our results, a positive association in all PF variables existed, while the sum of all the PF indicators produced an overall result at different stages of maturation. Based on the methodology of Mora-Gonzalez et al.,^39^ these findings suggest that the PF could be standardized and combined to form a global health indicator that might guide the decision-making of practitioners involved in children and adolescents’ PA and sport (such as physical education teachers or coaches). This indicator should be complemented by specific proposals and behaviour change strategies (depending on sex and maturity status) in each stage to increase daily PA and the acquisition of healthy habits.

There are some limitations to be considered when interpreting the present results. First, the study has a cross-sectional design, and factors, such as general activity, sedentary behaviour and diet, were not controlled. Furthermore, cause and effect could not be confirmed. Longitudinal studies comparing rural and active youth populations with a control group are therefore needed to confirm and clarify our findings. Second, the anthropometry test with a portable segmental analyser of multifrequency body composition was used in the field and not in the laboratory. However, although the reliability of the data is open to question, the very large sample means that this error softens.^40^ Third, although the maturity status was estimated using Tanner stages as in previous studies, the results should be interpreted with caution as they may be less accurate than other methods.

In conclusion, this study identified anthropometric parameters and performance in different PF tests amongst rural children and adolescents engaged in extracurricular sports according to maturation and sex. Absolute values of body composition and PF improved in pubertal children, but the percentile analysis reveal pubertal children to be less fit than prepubertal children, regardless of sex. In addition, body composition and PF showed a significant relationship with the maturation process. Therefore, this work provides further evidence to help policymakers, researchers and practitioners working with young people to promote more active and healthy behaviours among rural children and adolescents, taking into account factors, such as maturational status, chronological age, and sex.
